# Supplementary material for: A Novel Network Science and Similarity-Searching-Based Approach for Discovering Potential Tumor-Homing Peptides from Antimicrobials
Source: Antibiotics (Basel). 2022 Mar 17;11(3):401. doi: 10.3390/antibiotics11030401 (PMC8944733; doi:10.3390/antibiotics11030401)
Supplement: Supplementary file 1 [file antibiotics-11-00401-s001.zip › antibiotics-1601556-supplementary.pdf]

antibiotics

Table S1. CSN parameters of similarity threshold analysis.

Similarity threshold	Density	Communities	Modularity	Singletons	ACC
0.1	0.999	3	0.03	0	0.999
0.15	0.996	3	0.03	0	0.996
0.2	0.985	3	0.03	0	0.988
0.25	0.956	3	0.04	0	0.968
0.3	0.891	3	0.05	0	0.93
0.35	0.772	3	0.07	0	0.87
0.4	0.593	3	0.11	0	0.791
0.45	0.383	4	0.16	2	0.703
0.5	0.199	4	0.23	3	0.612
0.55	0.079	6	0.34	20	0.508
0.6	0.023	10	0.47	99	0.428
0.65	0.005	34	0.68	238	0.419
0.7	0.001	38	0.81	449	0.544
0.75	0	21	0.85	548	0.535
0.8	0	13	0.84	587	0.456
0.85	0	9	0.87	606	0.456
0.9	0	2	0.5	623	-
